# Assessing the Health Outcomes of the Food Access Pilot Project: An Evaluation of a Medically Supportive Food Support Program for People Living with HIV in Rural California Counties

**DOI:** 10.1007/s10461-022-03589-6

**Published:** 2022-02-05

**Authors:** Linda Yu, Amee Madura, Claudia Gil, Paul Hepfer, Kartika Palar

**Affiliations:** 1 School of Medicine, University of California, San Francisco (UCSF), San Francisco, CA USA; 2 The Health Trust, San Jose, CA USA; 3 Project Open Hand, San Francisco, CA USA; 4 Division of HIV, Infectious Diseases and Global Medicine, Zuckerberg San Francisco General, Department of Medicine, University of California, San Francisco (UCSF), 995 Potrero Ave, Building 80, Ward 84, Campus Box 0874, San Francisco, CA 94110 USA

**Keywords:** Food security, Food support, Medically supportive food, Rural, HIV

## Abstract

Food insecurity disproportionately affects rural communities and people living with HIV (PLHIV). The Food Access Pilot Project (FAPP) was a California state-funded program that provided home-delivered, medically supportive meals via online meal vendors to food-insecure PLHIV in three rural counties. We performed longitudinal, retrospective analyses of FAPP participant data (n = 158; 504 and 460 person-time observations for viral load and CD4 count, respectively) over 36 months from a Ryan White client management database. Pre-post analyses demonstrated increased prevalence of food security and CD4 ≥ 500 between baseline and 12 months. Population-averaged trends using generalized estimating equations adjusted for participant demographics demonstrated increased odds of viral suppression and CD4 ≥ 500, and increased CD4 count (cells/mm^3^) for every six months of program enrollment. Home-delivered, medically supportive meals may improve food security status, HIV viral suppression, and immune health for low-income PLHIV in rural settings.

## Introduction

Amongst people living with HIV (PLHIV), food insecurity—a state of limited access to nutritionally adequate food—is a well-documented driver of poor health outcomes, including lower viral load suppression and CD4 counts [1, 2], decreased adherence to antiretroviral therapy (ART) [1, 3, 4], increased acute care utilization [2], and increased mortality [5]. Research regarding the prevalence of food insecurity amongst PLHIV in rural areas is limited, though studies have found that over 50% of PLHIV in low-income, urban settings in California (CA) are food insecure [4], and that food insecurity disproportionately impacts rural communities [6]. This holds true in California, where the most food insecure counties in the state are located primarily within the agricultural Central Valley and in rural areas near the state’s northern border with Oregon [7]. Furthermore, PLHIV in rural areas have been found to experience delayed entry to care [8, 9] and increased HIV-related mortality [8, 10], which may be related to barriers such as unreliable transportation access [11–13], long distances to care [12], and limited access to providers with HIV expertise [13]. In light of these negative associations between food insecurity, rural residence and HIV clinical outcomes, improving nutritional food access and HIV care have become increasingly recognized priorities in both urban and rural settings.

Among people with HIV, diabetes and/or other chronic health conditions, programs providing medically supportive food—meals and groceries that meet basic nutritional needs and follow medical guidelines for disease prevention and management—have recently been shown to improve food security, mental health outcomes and chronic disease management [14, 15]. Medically supportive food programs, and in particular medically tailored meals, including those serving PLHIV [16], have also been associated with fewer emergency department visits [16, 17] and decreased inpatient and skilled nursing facility admissions [17, 18]. Several of these interventions have also been associated with reduced healthcare expenditures [16–18]. In a rapidly changing healthcare landscape that seeks to balance clinical outcomes with decreased healthcare spending, these studies lay the groundwork for the use of medically supportive meals in a holistic model of multimodal chronic disease management.

Thus far, many medically supportive food interventions have been implemented by large, non-profit organizations based in or near urban areas. Several prominent groups include Community Servings in Boston, Massachusetts, the Metropolitan Area Neighborhood Nutrition Alliance (MANNA) in Philadelphia, Pennsylvania, and Project Open Hand in San Francisco, California. In comparison, there is a relative paucity of medically supportive food interventions that address food insecurity in rural areas or that serve rural PLHIV, indicating the need for expanded approaches to provide nutrition services to PLHIV in rural communities.

The Food Access Pilot Project (FAPP) was a California state-funded program administered by The Health Trust, a non-profit organization based in the San Francisco Bay Area. It aimed to improve the health and nutrition of PLHIV in three rural CA counties (Humboldt, Napa and San Joaquin) by home-delivering individualized, medically supportive meals and pantry boxes via online vendors, while also connecting clients with telephone-based support from a registered dietitian (RD). As part of an external evaluation of this program, we assessed for changes in food security and HIV health outcomes, including viral load and CD4 count, amongst FAPP clients over the course of program participation. Based on prior published research, we hypothesized that FAPP participation would be associated with improvement in food security status and HIV clinical outcomes.

## Methods

### Study Design

We performed retrospective, longitudinal analyses of FAPP participant data from a Ryan White client management database, including food insecurity status and HIV clinical health outcomes, before and after program enrollment.

### Study Setting

The FAPP program was funded and initiated by the California State Office of AIDS (CDPH/OA) in response to concerns that food insecurity was a pressing area of need for PLHIV throughout the state—and particularly, in rural counties. For this pilot program, the CDPH/OA identified counties that lacked robust food bank utilization but had reliable case management services integrated with the AIDS Regional Information and Evaluation System (ARIES) (see Data Source below). While the goal of the FAPP program is to expand to all areas with need, these criteria allowed for the identification of pilot counties that had both a strong need for food support as well as a working infrastructure for patient referral and outcomes assessment. This led to the inclusion of Humboldt, Napa, and San Joaquin counties. Humboldt is over 300 miles north of the San Francisco Bay Area near the Oregon border and is the most rural of the three counties; Napa is in the greater Bay Area and is home to many lower-income communities despite its famous wineries; and San Joaquin is located within the agricultural Central Valley, which is predominantly rural but contains urban centers (e.g. Stockton).

### Program Population

To be eligible for FAPP program enrollment, clients were required to be eligible for and enrolled in Ryan White (have a diagnosis of HIV, receive an income under 500% of the Federal Poverty Level, and be uninsured or insured but still responsible for out-of-pocket costs such as copays), have a positive screen for food insecurity based on a two-item screener [19], and live in Humboldt, Napa, or San Joaquin counties. FAPP participation was the only eligibility criteria for individuals to be included in the secondary data analyses for the FAPP evaluation.

### FAPP Program Description

The FAPP program was implemented remotely by The Health Trust, a non-profit organization based in the San Francisco Bay Area, whose mission is to build health equity in Silicon Valley. It provides funding, direct services, and advocacy to improve health through food, housing, and chronic disease prevention and management.

As part of the FAPP program, clients could receive either home-delivered prepared meals or meal kits on a weekly basis, which were intended to provide approximately 30% of clients’ daily nutritional needs. Those who expressed further need could receive an additional monthly pantry box with non-perishable grocery items. All meals, meal kits, and pantry boxes were purchased by Health Trust staff for FAPP clients through online commercial meal vendors (HelloFresh: meal kits; Freshly: prepared meals; and Amazon: pantry boxes), and all vendors were vetted to ensure the quality and nutritional value of the food support and their ability to deliver to rural areas. Health Trust staff involved with day-to-day management of the FAPP program included a program coordinator and a RD.

Beginning in January 2017, local case managers in Humboldt, Napa, and San Joaquin counties referred food-insecure, Ryan White eligible HIV patients on a rolling basis to Health Trust staff for enrollment into the FAPP program. The Health Trust RD then contacted each new client via telephone to discuss their health needs, medical comorbidities, and food preferences in order to select the optimal format and contents of the food that would be provided. For example, clients with limited kitchen access or physical limitations hindering food preparation were able to choose the prepared meals option, while those who preferred to cook their own meals could choose to receive meal kits. Throughout the program, the RD provided nutritional counseling and support as needed, and clients were able to switch between meals and meal kits if they wished. Health Trust staff also routinely checked in with clients to offer support and address any issues with food delivery or program participation.

All meals, meal kits and pantry boxes were provided at no cost to program participants, and meal deliveries continued until a recipient chose to withdraw or could not be contacted by program staff or county case managers. Due to rolling enrollment, the duration of program enrollment amongst clients ranged from several months to several years.

### Data Source

With approval from the CDPH/OA, which funded both the FAPP program and the subsequent program evaluation, The Health Trust provided the evaluation team with de-identified data from ARIES, a statewide HIV/AIDS client management database funded and overseen by the CDPH/OA. ARIES tracks and supports the coordination of care provided by Ryan White-funded service providers for PLHIV covered by the Ryan White HIV/AIDS Care Act. When PLHIV access Ryan White clinical care or ancillary services, data from these encounters (e.g. lab results for viral load and CD4 count) are entered into ARIES and viewable to other Ryan White providers. All HIV health outcomes in ARIES used for this analysis involved lab data resulting from healthcare visits conducted as part of usual HIV care received by the client, i.e. not for research purposes or related to the FAPP program. During the program, Health Trust staff also entered data on client demographics and food insecurity into ARIES for internal assessment purposes. The evaluation team received de-identified ARIES data for FAPP participants on health and program variables dated from January 2017 through March 2019.

#### Dataset Creation

**Fig. 1:**
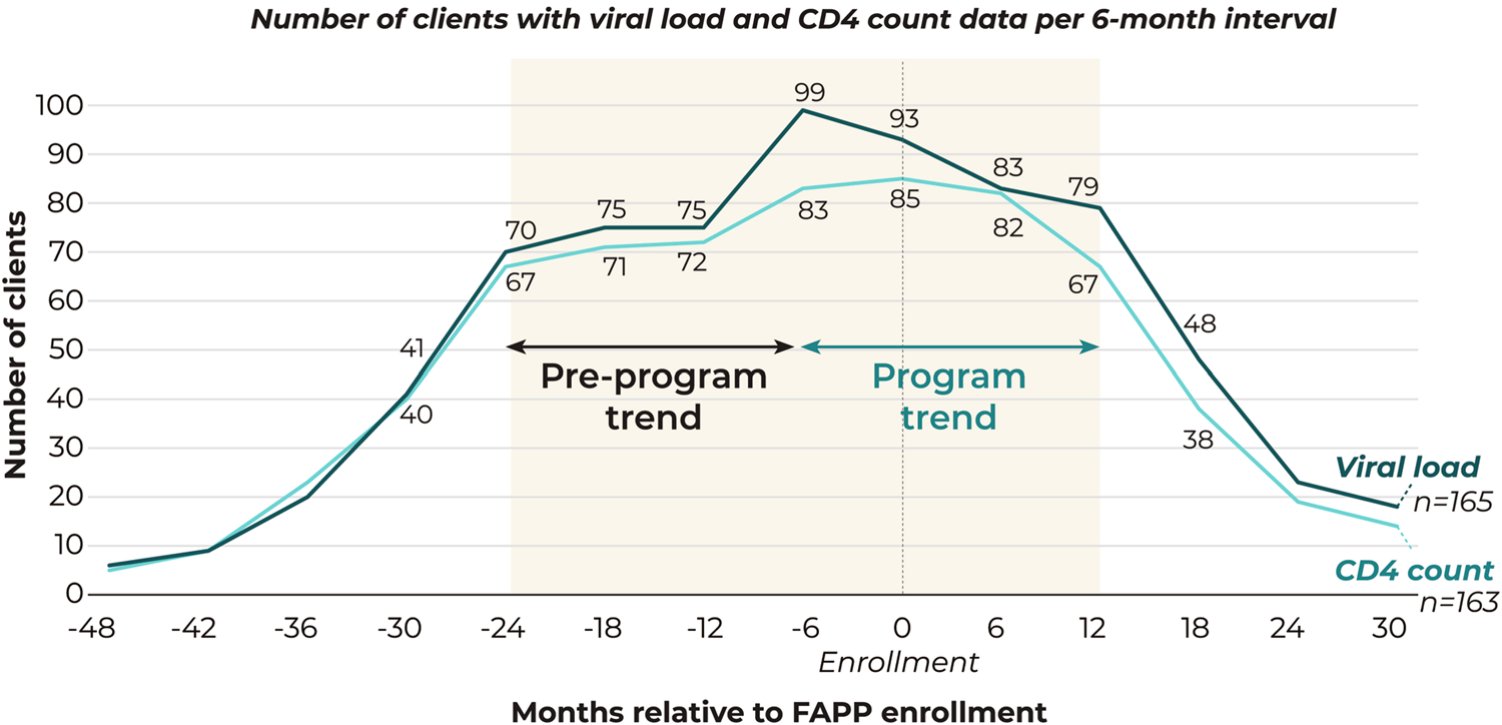
Viral load and CD4 count data completion in ARIES, per 6-month interval

Given that HIV outcomes data in ARIES came from healthcare encounters and not research assessments, HIV viral load and CD4 count data were not available at pre-set or prescribed intervals in relation to the FAPP program. Instead, they were distributed unevenly, with gaps of various intervals between consecutive tests. Given this dispersed data structure, as well as rolling enrollment into the FAPP program, we created an analytic dataset by first processing each client’s data relative to their own enrollment date. ARIES data was available for the greatest number of clients during a 36-month window from 24 months prior to enrollment to 12 months after, with significant drop-off in data availability outside of this time frame (Fig. 1). Based on Centers for Disease Control (CDC) guidelines for PLHIV to check their CD4 count every 3–6 months and HIV viral load every 4–6 months [20], we created a longitudinal data set based on 6-month intervals, using the date of enrollment as the index date, over the 36-month period defined above. We then identified the most recent lab result available within each 6-month period for each outcome and assigned this value as the data point for that time interval. If no value was available in a 6-month time frame, it was set as missing. Baseline values were set as the most recent value within six months prior to program enrollment.

### Outcomes

We used data from ARIES to assess the following outcomes.

#### Food insecurity

Food insecurity was assessed by Health Trust staff at program enrollment and 12 months after enrollment using two questions [19] adapted from the USDA Household Security Survey Module [6] related to the adequacy of and uncertainty around household food supply: (1) Within the past 30 days, I worried whether my food would run out before I got money to buy more, and (2) Within the past 30 days, the food I bought just didn’t last and I didn’t have money to get more. If the client answered “often” or “sometimes” true to either question, they were considered food insecure.

#### HIV Health Outcomes

##### HIV Viral Load (VL)

We assessed viral suppression as having a viral load below 200 copies/mL (VL < 200), per CDC definitions [21], which suggests low viral activity and decreased risk of HIV-related illness. We also assessed undetectable viral load, which indicates non-transmissibility of the HIV virus [21]. The threshold for undetectable viral load varies based on the specific test used and is usually between 20–50 copies/mL. Since we did not have information about the specific tests used to generate the viral load data in ARIES, we chose to define an undetectable viral load as a level below 40 copies/mL (VL < 40), a common cut-off [22].

##### CD4 Count

We determined whether individuals had a CD4 count greater than or equal to 500 cells/mm^3^ (CD4 ≥ 500), a standard clinical marker of healthy immune function [23, 24]. We also assessed for changes in CD4 count as a continuous variable.

### Analysis

#### Pre–Post Analysis

First, we assessed paired outcomes at baseline and 12 months amongst clients with data available at both time points (n = 87 for food insecurity; n = 45 for VL; n = 33 for CD4 count). Baseline values were identified as the outcome closest to enrollment prior to enrollment within a 6-month window. The 12-month values were the outcomes recorded closest to 12 months, between 6 to 12 months of enrollment. If a participant did not have an outcome value in both of these time ranges, they were excluded from the paired analysis. We used McNemar’s test to assess changes in the proportion of clients achieving food security, viral suppression, undetectable viral load and CD4 ≥ 500. A pre-post difference was statistically significant at α = 0.05 using the Exact McNemar’s significance probability.

#### Population-Averaged Trends Analysis

Given the small sample sizes available for the paired pre-post analysis described above, we then used generalized estimating equations to estimate population-averaged trends in viral suppression, undetectable viral load, CD4 ≥ 500, and continuous CD4 count at 6-month intervals between baseline and the first 12 months of program enrollment, spanning 18 months total (“program trends”). Logistic regression with GEE was used to assess dichotomous outcomes and linear regression with GEE was used to assess the continuous outcome, with the time interval as the primary explanatory variable. All models were adjusted for age, gender, race/ethnicity and county to address potential confounding by demographic or geographic differences, and used robust standard errors. A sensitivity analysis was conducted including “time on ART” as an additional covariate. This variable was not included in the main analysis given that about one-third of participants were missing data on their duration of ART.

Finally, we took advantage of the existence of the longitudinal pre-program data 24 months prior to enrollment up until 6 months prior to enrollment (i.e. the farthest time point before the baseline interval), in order to assess population-averaged trends in clinical outcomes in the period immediately prior to the baseline (“pre-program trends”). The purpose of this analysis was to address the possibility that any program trends in viral load or CD4 count we observed could be the result of pre-existing trends.

Stata 16 (Stata Corp., College Station, TX, USA) was used for all analyses. The UCSF IRB designated this program evaluation utilizing de-identified secondary data as a quality improvement initiative rather than human subjects research, and so did not exercise direct oversight of the evaluation. The release of the de-identified secondary data was approved by CDPH/OA, and the evaluation team practiced rigorous procedures to protect the confidentiality of participant data. As part of FAPP program enrollment, all participants provided written consent to have their ARIES data shared for evaluation purposes.

## Results

### Sample Characteristics

We analyzed the demographics and health characteristics of 191 FAPP participants enrolled between January 2017 and March 2019 (Table 1). At enrollment, 67.5% of participants were male and 41.9% were non-Hispanic White. Median age was 52 years (IQR 41, 58). Almost one-third (28.8%) reported living in temporary or unstable housing. Most clients were long-term survivors of HIV; the median length of time on antiretroviral therapy was 9.48 years. In the 6 months prior to FAPP enrollment, amongst clients with available data (n = 93 for VL; n = 85 for CD4), 80.6% were virally suppressed (VL < 200), 69.9% of clients had an undetectable viral load (VL < 40), and 51.8% had a CD4 count ≥ 500. Amongst all enrolled clients, 63.9% were enrolled for 12 or more months.

**Table 1:** FAPP participant demographics and clinical characteristics at baseline, by county (n = 191)

Characteristic		County		
	Overall (n = 191)	San Joaquin (n = 93)	Humboldt (n = 62)	Napa (n = 36)
*Demographics*				
Gender, No. (%)^a^				
Men	129 (67.5)	53 (57.0)	47 (75.8)	29 (80.6)
Age, median (IQR)	52 (41, 58)	52 (41, 58)	54 (47, 59)	46 (37, 53.5)
Race/ethnicity, No. (%)				
Non-Hispanic white	80 (41.9)	24 (25.8)	47 (75.8)	9 (25.0)
Non-Hispanic black	49 (25.7)	43 (46.2)	5 (8.06)	1 (2.78)
Hispanic	51 (26.7)	22 (23.7)	6 (9.68)	23 (63.9)
Other	9 (4.71)	4 (4.30)	4 (6.45)	1 (2.78)
Unknown	2 (1.05)	0 (0.00)	0 (0.00)	2 (5.56)
Sexual orientation, No. (%)				
Heterosexual	106 (55.5)	63 (67.7)	29 (46.8)	14 (38.9)
Lesbian, Gay, Bisexual	77 (40.3)	27 (29.0)	31 (50.0)	19 (52.8)
Unknown/Declined to state	8 (4.19)	3 (3.23)	2 (3.23)	3 (8.33)
Primary language: English	169 (88.5)	88 (94.6)	59 (95.2)	22 (61.1)
Education, No. (%)				
Less than high school GED	43 (22.5)	31 (33.3)	12 (19.4)	0 (0.00)
High school GED or higher	94 (49.2)	49 (52.7)	44 (71.0)	1 (2.78)
Unknown	51 (26.7)	10 (10.8)	6 (9.68)	36 (97.2)
Housing, No. (%)				
Stable housing	133 (69.6)	63 (67.7)	48 (77.4)	22 (61.1)
Temporary housing	38 (19.9)	23 (24.7)	4 (6.5)	11 (30.6)
Unstable housing	17 (8.90)	5 (5.38)	9 (14.5)	3 (8.33)
Unknown	3 (1.57)	2 (2.15)	1 (1.61)	0 (0.00)
*Health characteristics*				
Years on ART therapy: median (IQR)^d^	9.48 (4.95, 12.9)	8.07 (5.30, 11.8)	9.80 (2.36, 13.7)	9.75 (5.79, 14.7)
Unknown, No. (%)	55 (28.8)	35 (37.6)	16 (25.8)	4 (11.1)
HIV viral load status, No. (%)^b^				
Virally suppressed (VL < 200)^d^	75 (39.3)	30 (32.3)	25 (40.3)	20 (55.6)
Undetectable viral load (VL < 40)	65 (34.0)	24 (25.8)	24 (38.7)	17 (47.2)
Unknown	98 (51.3)	55 (59.1)	30 (48.4)	13 (36.1)
CD4 count, No. (%)^c^				
CD4 ≥ 500	44 (23.0)	14 (15.1)	18 (29.0)	12 (33.3)
CD4 count, mean (SD)	562 (360)	475 (365)	639 (374)	574 (318)
Unknown	106 (55.5)	62 (66.7)	30 (48.4)	14 (38.9)

^a^County percentages are relative to the county total

^b^Viral load is measured in copies/mL

^c^CD4 count is measured in cells/mm^3^

^d^IQR = interquartile range, VL = viral load

### Pre–Post Analysis

Of the 191 participants in the study, a limited subset of people had paired data on food insecurity (n = 87), viral load (n = 45) and CD4 count (n = 33) at baseline and 12 months. On average, participants from San Joaquin county were less likely to have paired data compared to those from Humboldt and Napa. There were no significant differences in terms of age, gender, and race between those with paired data and those without.

#### Food Insecurity

At baseline, all FAPP clients screened positively for food insecurity, i.e. none were food secure. After 12 months, the proportion of clients reporting food security increased significantly from 0% to 62.1% (p < 0.001) (Table 2).

**Table 2:** Food security, viral load, and CD4 count at baseline and 12 months after enrollment

Health outcome	No. of clients with paired data	Baseline^a^	12 months^b^	p-value^c,d^
*Food security*				
Food insecure, No. (%)	87	0 (0%)	54 (62.1%)	< *0.001*
*HIV health*				
Viral suppression (VL < 200), No. (%)	45	40 (88.9%)	43 (95.6%)	0.375
Undetectable viral load (VL < 40), No. (%)	45	34 (75.6%)	38 (84.4%)	0.388
CD4 ≥ 500, No. (%)	33	16 (48.5%)	22 (66.7%)	*0.031*

^a^Baseline indicates outcome closest to enrollment within the 6-month window prior to enrollment

^b^12 months indicates outcome recorded closest to 12 months, between 6 to 12 months of enrollment

^c^p-values calculated using McNemar’s test

^d^Italicized p-values are statistically significant at p < 0.05

#### HIV Viral Load

Amongst clients with data at baseline and 12 months (n = 45), we observed an increase in the proportion of individuals with viral suppression (88.9% to 95.6%, p = 0.375) and undetectable viral load (75.6% to 84.4%, p = 0.388) at 12 months, though these results were not statistically significant (Table 2).

#### CD4 Count

Amongst clients with data at baseline and 12 months (n = 33), we observed an increase in the proportion of individuals with CD4 ≥ 500 (48.5% to 66.7%, p = 0.031) (Table 2). All 16 clients with CD4 ≥ 500 at baseline maintained this level at 12 months. Of the 17 clients with CD4 < 500 at baseline, 6 (35.3%) had a CD4 count ≥ 500 at 12 months.

### Population-Averaged Trends Analysis

Of the 191 participants in the study, 158 clients had data within the 36-month time frame and were included in the analytic data set. These clients consisted of a greater proportion of clients from San Joaquin and a smaller proportion of clients from Humboldt, but there were no significant differences in terms of age, gender, or race compared to the group of excluded clients. On the whole, the analytic data set consisted of 504 person-time observations for viral load (pre-program trend: 249, program trend: 255) and 460 person-time observations (pre-program trend: 226, program trend: 234) for CD4 count.

#### HIV Viral Load

For every 6 months of FAPP enrollment, there were 91.2% increased odds (aOR = 1.91, p = 0.002) of viral suppression (Table 3). There was no identifiable trend prior to enrollment. There were also no identifiable trends in the odds of undetectable viral load during or preceding the FAPP program.

**Table 3:** Viral load and CD4 count trends prior to and after FAPP enrollment

	Pre-program trend			Program trend		
Health outcome	aOR	95% CI	p-value	aOR	95% CI	p-value^c^
*HIV viral load*^a^						
Viral suppression (VL < 200)	1.13	0.810, 1.58	0.472	1.91	1.26, 2.91	*0.002*
Undetectable viral load (VL < 40)	1.17	0.910, 1.50	0.224	1.14	0.824, 1.57	0.435

	Pre-program trend			Program trend		
	aOR	95% CI	p-value	aOR	95% CI	p-value
*CD4 count*^b^						
CD4 ≥ 500	1.12	0.958, 1.30	0.158	1.43	1.15, 1.77	*0.001*

	β	95% CI	p-value	β	95% CI	p-value
Continuous CD4 count (cells/mm^3^)	16.5	− 5.28, 38.3	0.138	32.2	7.74, 56.7	*0.010*

*aOR* adjusted odds ratio, *CI* confidence interval

^a^Sample sizes, viral load: VL pre-program trend (249 person-time observations, 127 unique ID’s); VL program trend (255 person-time observations, 140 unique ID’s)

^b^Sample sizes, CD4: CD4 pre-program trend (226 person-time observations, 117 unique ID’s); CD4 program trend (234 person-time observations, 137 unique ID’s)

^c^Italicized p-values are statistically significant at p < 0.05

#### CD4 Count

For every 6 months of FAPP enrollment, there were 42.8% increased odds (aOR 1.43, p = 0.001) of CD4 ≥ 500 (Table 3). Prior to enrollment, there was no statistically significant trend. In the linear regression analysis, there was an increase in CD4 count of 32.2 cells/mm^3^ (p = 0.010) for every 6 months of enrollment. Prior to enrollment, there was an increase in CD4 count of 16.5 cells/mm^3^ which was not statistically significant (p = 0.138).

In the sensitivity analysis including time on ART as a covariate, the adjusted odds ratios for VL < 200 and CD4 ≥ 500 were slightly increased, while all other trends were preserved.

## Discussion

In this study investigating the health outcomes associated with weekly deliveries of medically supportive meals, we found that FAPP participation was associated with significant increases in the prevalence of food security and the odds of achieving viral suppression and healthy immune function over time. These results are consistent with previously published studies documenting the positive health outcomes associated with medically supportive meals, while also extending those findings to a real-world program serving PLHIV in rural settings.

Several past trials of medically supportive meals have included PLHIV within their study population [14, 16, 18], but few have reported changes in clinical HIV outcomes. A recent pilot study of medically supportive meals based in the San Francisco Bay Area found that program participation was associated with significant improvement in ART adherence from 47 to 70% [14], suggesting one possible pathway linking food security to improved HIV outcomes. However, this study did not report any laboratory measures and was unable to assess for changes in viral load or CD4 count. Other literature has largely focused on outcomes such as healthcare utilization [18] or healthcare costs [16]. Our evaluation is thus one of the first to report positive associations between a medically supportive meal program and HIV clinical outcomes.

Many programs that provide medically supportive meals have been implemented by non-profit organizations in urban centers that serve a local region, which may present accessibility challenges for individuals living outside of these areas. In a recent study conducted with Community Servings in Boston, clients needed to live within 100 km (approximately 60 miles) in order to participate [18]. In the San Francisco Bay Area, Project Open Hand serves residents of San Francisco and Alameda counties [25], while MANNA in Philadelphia serves nine counties in the Greater Philadelphia Area and Southern New Jersey [26], approximately a radius of 30–40 miles. As organizations look ahead toward expanding their meal delivery services, it becomes imperative to design food support programs capable of serving both urban and rural populations, whether through building and strengthening local capacity within counties, or through exploring mechanisms that expand delivery radius from an urban center.

The FAPP model has several key features, including the use of online meal vendors, involvement of a registered dietitian, and integration of local case management infrastructure, which speak to both the potential benefits and drawbacks of this program model for rural—and urban—underserved areas. A program report detailing results from semi-structured interviews with FAPP stakeholders [27] suggested that the wide-reaching delivery networks of online meal vendors allowed the program to overcome barriers to rural delivery, which was perceived as a main benefit of the FAPP model. Additionally, the inclusion of multiple vendors supplying different forms of food support (meal kits, prepared meals, and pantry boxes) allowed the program to serve a diverse range of client needs. However, stakeholders also expressed concerns that online meal vendors—while able to provide food support that was generally in line with medical guidelines—were limited in their ability to tailor meals for patients with more specific or restrictive dietary needs, such as those requiring a renal diet. There were also questions about the sustainability of relying on online meal vendors in the context of a shifting commercial landscape, such as concerns about cost and the alignment of missions between non-profit and for-profit organizations.

Program stakeholders previously reported that centralized RD support was among the FAPP program benefits [27]. The RD, based at the Health Trust, was able to provide individualized nutritional counseling and support over the phone to patients in under-resourced rural counties that did not have other options for local nutrition education support. The RD also played a critical role in providing oversight of the nutritional content of the meals, meal kits, and pantry boxes in order to ensure their medical appropriateness**.** However, direct RD involvement tends to also raise the cost of food programs and may not be strictly necessary for all activities; finding alternatives may help to make the program more sustainable. Finally, the program model leveraged the expertise and existing relationships of county case managers with their clients, which were critical for program recruitment and client retention. Counties without strong case-management programs—which may also have a higher need for programs like the FAPP—may not be able to equally participate in and benefit from this type of program model. While our results indicate that the FAPP is promising, key practical considerations need to be addressed for programs utilizing similar models to sustainably improve the health for PLHIV in rural areas on a wider scale.

The results of this study should be interpreted in light of several limitations. First, this study did not include a control group, and thus we cannot rule out that the improvements we observed in viral load and CD4 count may be due to external factors rather than participation in the program itself. For instance, we cannot rule out the possibility that these results were related to ongoing, statewide efforts to decrease HIV transmissions and eliminate HIV-related deaths, or local efforts to improve HIV care in the three counties. Nonetheless, it is still plausible that these positive health outcomes are in part related to FAPP participation, as our data suggests almost no statistically significant pre-program trends, and previous qualitative interviews with stakeholders suggested perceived changes in client health outcomes. Next, our analysis was limited by a high degree of missing viral load and CD4 count data in the ARIES database—approximately 20% of clients had paired data available for the pre-post analysis, and 80% for the population-averaged trends analysis. There may be many reasons for these missing data, including patients missing care appointments, incomplete or delayed reporting of clinical data by providers, or participants visiting non-Ryan White-funded providers whose data are not reported to ARIES. It is possible that patterns of missing data could be related to patterns of change in viral load and CD4 count (e.g. if clients with more missing data were unable to access care due to more severe illness), which could bias the results away from the null. Finally, this study may not be generalizable to other rural counties within or outside of California, as the counties chosen for this program were required to have robust case management services in order to facilitate client recruitment, to minimize loss to follow-up, and for logistical coordination. This may not be the case for all counties and could potentially pose barriers to implementation.

This evaluation offers insight into the feasibility and potential impact of a food support intervention designed specifically to serve a rural population with HIV. FAPP participation was associated with improvement in food security status, HIV viral suppression and immune health, suggesting a promising role for home-delivered, medically supportive meals for PLHIV in rural counties. These findings should be tested using more rigorous study designs including a control group to assess program impact. The FAPP model utilizes several innovative features to overcome rural barriers to food delivery and may offer important insight to inform the design and implementation of medically supportive food programs for vulnerable populations—both urban and rural—moving forward.

## Data Availability

The participant data in this study is part of a health services database governed by the CDPH/OA shared with the study team specifically for the purposes of program evaluation. We do not have permission to share this data publicly unless permission is granted by the CDPH/OA.

## Code Availability

Stata 16 (Stata Corp., College Station, TX, USA) was used for all analyses. We are willing to share our study code upon request.
